# Early Blood Analysis and Gas Exchange Monitoring in the Canine Neonate: Effect of Dam’s Size and Birth Order

**DOI:** 10.3390/ani12121508

**Published:** 2022-06-09

**Authors:** Brenda Reyes-Sotelo, Asahi Ogi, Patricia Mora-Medina, Chiara Mariti, Adriana Olmos-Hernández, Ismael Hernández-Ávalos, Adriana Domínguez-Oliva, Marcelino Evodio Rosas, Antonio Verduzco-Mendoza, Angelo Gazzano

**Affiliations:** 1 Master in Science Programme “Maestria en Ciencias Agropecuarias”, Xochimilco Campus, Universidad Autónoma Metropolitana, Ciudad de México 04960, Mexico; breyess_20@yahoo.com.mx; 2 Neurophysiology, Behavior and Animal Welfare Assessment, Xochimilco Campus, Universidad Autónoma Metropolitana, Ciudad de México 04960, Mexico; cnrverduzco@hotmail.com; 3 Department of Veterinary Sciences, University of Pisa, 56124 Pisa, Italy; asahi.ogi@vet.unipi.it (A.O.); chiara.mariti@unipi.it (C.M.); 4 Facultad de Estudios Superiores Cuautitlán, Universidad Nacional Autónoma de México (UNAM), Cuautitlán 54714, Mexico; mormed2001@yahoo.com.mx (P.M.-M.); mvziha@hotmail.com (I.H.-Á.); rosasgme@hotmail.com (M.E.R.); 5 Division of Biotechnology—Bioterio and Experimental Surgery, Instituto Nacional de Rehabilitación—Luis Guillermo Ibarra Ibarra (INR-LGII), Ciudad de México 14389, Mexico; adrianaolmos05@gmail.com

**Keywords:** puppy welfare, hypoxia, size, physiological blood profile, asphyxia, whelping

## Abstract

**Simple Summary:**

The complications that are observed during parturition are events that affect the vitality of the newborn and can also compromise their health by predisposing them to fetal hypoxia, increasing newborn mortality. Blood gas analysis to measure the main biomarkers associated with hypoxia evaluates the physiological and metabolic alterations derived from this state, and these could help identify if said markers respond to maternal or neonatal causes. This study aimed to assess the effect of the dam’s size, the birth order, and the presentation of blood gas alterations. Recognizing if these elements are intertwined may enhance newborns’ life expectancy by enabling the planning of a perinatal protocol to avoid serious metabolic consequences that are derived from prolonged hypoxia.

**Abstract:**

In canines, size at birth is determined by the dam’s weight, which would probably affect the newborn’s viability due to litter size and birth order. Fetal hypoxia causes distress and acidemia. Identifying physiological blood alterations in the puppy during the first minute of life through the blood gas exchange of the umbilical cord could determine the puppy’s risk of suffering asphyxiation during labor. This study aimed to evaluate the effect of the birth order and dam’s size during spontaneous labor and the alterations during the first minute of life. The results indicate that the dam’s size and the birth order have considerable physiological and metabolic effects in the puppies, mainly in birth order 1 (BO1) in small-size dogs, while in the medium size, the last puppy presented more alterations, probably because of a prolonged whelping which could have fostered hypoxic processes and death. Likewise, with large-size dogs, intrapartum asphyxiation processes were registered during the first minute of life in any birth order.

## 1. Introduction

Birth represents a great challenge for neonatal survival in a new environment with different conditions. The transition from fetal to neonatal life involves an efficient multisystemic adaptation where the most critical change is related to the start of breathing [1,2]. Parturition induces significant physiological and adaptative events for mammal species due to the cardiovascular, respiratory, and thermoregulation changes the newborn will confront in the postnatal period [3]. During parturition, transient hypoxia periods are present in the fetus due to the uterine contractions and the mechanical pressure inherent to birth, causing a decrease in blood flow and placental perfusion, compromising gas interchange in the fetus [4]. The state of transitory fetal hypoxia that the pups endure during the perinatal period is part of the natural process of birth [5]. Recognizing a hypoxic process in the newborn is critical for its survival; however, the absence of evident signs in pups, such as hyperventilation, limits its identification [6].

To obtain the physiological stability profile of the newborn, parameter assessment related to the morphological characteristics of the umbilical cord, such as edematous, hemorrhagic, congested or ruptured cord [7] and blood metabolites is suggested. Lactate plays a central role in diagnosing neonatal and fetal distress when the oxygen supply is interrupted, tissular oxygen deprivation develops, and acids start to accumulate, developing a state of acidemia [8].

In the beginning, James and collaborators [9] found that evaluating blood gases in the umbilical cord added fetal hypoxic stress data and provided relevant information during the perinatal period. Moreover, they give a panorama of the acid-base state of the neonate at the moment of birth, when the female blood circulation is interrupted by the occlusion or tear of the umbilical cord [10,11]. Recently, gasometric assessment in the field of neonatal veterinary is an important tool to assess newborn health. The umbilical cord blood analysis allows the evaluation of the concentrations of glucose, lactate, partial pressure of oxygen (pO_2_), partial pressure of carbon dioxide (pCO_2_), pH, hematocrit, sodium, potassium, and ionized calcium [12]. The blood evaluation of the umbilical cord through gasometry allows the obtention of a physiometabolic profile to guarantee neonatal health [5].

A mature fetus can have abundant reserves of glycogen, making him more tolerant to intermittent periods of hypoxia. However, it is considered that the morphological variability in dogs could affect fetal development [2,13,14,15]. For newborn puppies, the presence of other factors, such as maternal care [16,17], uterine inertia [18,19], and prolonged labor predisposes them to hypoxia and Type II stillborn (SB) [20,21,22,23]. Type II stillborn (SB) is considered to be an effect that is associated with the oxytocinergic system of the dam and its regulation. Hypoxia and SB could have a negative effect on the postnatal adaptation or survival of the neonate [23,24] and it can be reflected in an altered blood physiological profile.

The challenges of the parturition process, along with its risk factors might determine the proportion of the liveborn pups (LBP) in comparison to the stillbirths (SB), as well as the viability of the former [25,26,27]. Therefore, the morphological variability in canines could be associated with the dam’s size and weight [28], and these factors might affect the pups’ weight and physiological state at birth.

In pigs, it has been reported that the integration of physiological, neurological, and behavioral factors at birth to recognize hypoxia and its repercussions has been of great importance because of the high incidence of stillborn piglets [29]. According to a study on dogs, this is also related to litter size and birth order, mainly affecting those belonging to the last quarter of the litter [26]. The major risk that has been identified is placental insufficiency and a greater risk of suffering intrauterine hypoxia [27,30,31]. Because of the aforesaid, birth order has been highlighted in other species, such as pigs, as being an indicator of survival [32,33,34]; however, in canines, research is still limited and has not included other risk factors, such as the dam’s size, which is related in this species with the size of the litter. This study aims to evaluate the effect of the dam’s size and birth order on the physiological responses of the canine neonate during the first minute of life.

## 2. Materials and Methods

### 2.1. Infrastructure, Animals, and Management

#### 2.1.1. Infrastructure

A network of 5 veterinarian clinics located in Mexico City was gathered to recruit pregnant dogs from January to June 2019. A prenatal control was held from the 25th day of pregnancy to the first 24 h post-birth.

#### 2.1.2. Study Population

A total of 58 young multiparous female dogs (2 to 4 births) were recruited. The inclusion criteria were, clinically healthy female dogs, updated vaccination/deworming schedule, fed with commercial formulas, no history of reproductive problems, and radiographic and ultrasound evaluation that ensured eutocic delivery. The exclusion criteria included, primiparous females, a background of dystocia or uterine infections, the presence of type I stillbirths, fetal malformations, the use of delivery inducers or accelerators, females with 8 and 9 body condition scores (obese) according to the WSAVA scale [35], and animals with extremely aggressive behavior. Brachycephalic and gigantic breeds were excluded from the study due to their high incidence of dystocia [22]. The 58 pregnant females were classified in 3 size categories according to their weight: small (<10 kg, *n* = 18); medium (11–25 kg, *n* = 20); and large (26–45 kg, *n* = 20) [15,36,37].

#### 2.1.3. Clinical History

The females’ clinical history included the following data: age, weight, feeding, prevention medicine conditions, and a description of the environment in which they inhabited. All the information was recorded in the Qvet**^®^** Ed. Professional 2016 database for veterinary clinics.

#### 2.1.4. Pregnancy Diagnosis

The gestation diagnosis was confirmed between the 24 and 28 days post-insemination for each female. The fetal structures and cardiac activity were detected inside the gestational sacs. Using Logiq 400 MD (General Electric**^®^,** Yokohama, Japan) ultrasound, probable birth dates were established with a 3.5 MHz convex transductor. The monitoring of fetal maturation and vitality was performed in the 40–50 days of progression when the fetal structure is completely defined, which allows the early identification of pyometra cases, type I stillbirths, and malformations. Afterward, a simple abdomen radiographic study was performed after day 45 of gestation once the bone calcification of the fetus had been reached to discard early fetal-dam dystocia, as well as to provide evidence of cephalopelvic disproportion, which would imply the need for cesarean-section [31], another motive to exclude the dog from the study. On day 60 of pregnancy, the female dogs were assessed by ultrasound to corroborate heartbeat and biparietal fetal diameter. The delivery monitoring was performed through a Sino-Hero**^®^** vital signs monitor, model S80Vet (Guangdong, China), to evaluate the dam’s physiological parameters. Clinical signs in the intrapartum were observed, such as anorexia, distress, and nesting behavior.

### 2.2. Puppies and Evaluated Variables during the First Minute of Life

The number of puppies, considering those born alive and dead (stillbirths type II and antepartum deaths—Type I), according to the female dog’s size, birth order, and physiological blood profile (of the sample taken from the umbilical cord) was registered for every birth.

However, a total of 310 live-born puppies (LBP) were studied in this stage, distributed in 3 categories according to the dam**’**s size: small *n* = 75, medium *n* = 102, large *n* = 133, considering the following criteria:

(a) Live-born puppies (LBP): neonates who presented breathing and heart frequency during the first minute of life;
(b) Stillborn Puppies (SBP): fetuses classified as dead intrapartum (type II) presented the same appearance as their litter partners, except for the absence of breathing;
(c) Antepartum deaths (type I): those with brown-grayish discoloration due to the initial state of mummification; and the most advanced cases in a clear dehydration state and with hair loss.

The fetuses (type I) and Stillborn (type II) were excluded from the study.

#### 2.2.1. Blood Physiological Profile

##### Blood Sampling

Veterinarian staff took blood samples from LBP immediately after birth in less than 10 s. Hemostasis was performed in the distal portion of the umbilical cord with a clamp. Subsequently, the index finger and thumb were placed on the base of the abdominal insertion of the umbilical cord and moved up to three centimeters from its distal portion. Once the turgidity and dilatation of the vessel were observed, blood sampling was obtained from the vein. The identification of the umbilical vein was according to its superficial location and appearance, where the vein can be recognized as a thick cyanotic blood vessel, while the artery is deeper with a more intense-bright red color and a smaller caliber. A total of 0.3 mL of venous blood from the umbilical cord was obtained with a tuberculin syringe with a 26G needle that was impregnated with lithium heparin to prevent coagulation and alteration of the sample values. A volume of 150 μL was processed through the analyzer of blood critical variables GEM Premier**^®^** 3000 (Instrumentation Laboratory Diagnostics; Lexington, KY, USA/Milano, Italy) to obtain the values of metabolites; glucose (mg/dL); lactate (mg/dL); blood gases pCO_2_ (mmHg); pO_2_ (mmHg); acid-base balance pH; HCO_3_^−^ (mmol/L) and base excess (BE) (mEq/L); Ca^2+^ (mmol/L); and hematocrit (Htc %). The physiological blood profile was evaluated for all the LBP. The pups did not receive any resuscitation before blood sampling so as not to interfere with gas exchange.

#### 2.2.2. Birth Order

The birth order was registered for every puppy (LBP), obtaining a classification by the dam’s size. For the small size, the total number of puppies by litter was 5 (BO1–BO5); for the medium size, 7 puppies (BO1–BO7); and for the large-size, 9 puppies (BO1–BO9).

### 2.3. Statistical Analysis

Data were organized in means ± SE. The effect of the dam’s size category (small, medium, and large) and birth order, as well as the interaction between these factors were obtained using variance analysis (ANOVA) by the GLM procedure (General Linear Model) [38] under the following model:

Metabolites_ijk_ = µ + *T_i_* + *BO_i_(T_i_BO_i_) + e_ijk_*

Metabolites = pH, pCO_2_, pO_2_, glucose, Ca^2+^, lactate, hematocrit, HCO_3_^−^, EB

µ = General mean

***T_i_*** = Fixed effect size (small, medium, large)

*BO**_i_*** = birth order 1, 2, 3, 4…e = error

The multiple comparison of means was performed with the Tukey test.

A Pearson rank test was used to establish the correlation between physiologic blood variables (pH, pCO_2_, pO_2_, glucose, lactate, HCO_3_^−^, Ca^2+^, hematocrit) and the dam’s size.

### 2.4. Ethics Note

The study was held on private property female dogs, and informed consent from every owner was obtained to gather the data. During the study, all the animals were managed according to the guidelines and rules of the Mexican Official Norm NOM-062-ZOO-1999, technical specifications for the production, care and use of laboratory animals, besides those of the competence of the field of applied ethologic studies [39].

The experimental protocol with number CAMCA.32.18 was approved by the Commission of the Master’s in Agriculture and Livestock Sciences of the Universidad Autonoma Metropolitana, Xochimilco, Mexico City.

## 3. Results

The results of this project included the variable of birth order (BO) in female dogs of different sizes and its relation to the litter size. The BO was classified according to the size as follows: for small-size bitches (BO1–BO5), medium-size (BO1–BO7), and large-size (BO1–BO9).

### 3.1. Physiological Parameters According to Birth Order in Canine Neonates (LBP) from Small-Size Female Dogs

Table 1 shows that, regarding the acid-base and energetic balance, the puppies did not present significant differences in lactate (*p* = 0.12); glucose (*p* = 0.20); pH (*p* = 0.53); HCO_3_^−^ (*p* = 0.35); and BE (*p* = 0.16) levels in the blood, among the groups (BO1, BO2, BO3, BO4, BO5). However, in Table 2, it is observed that blood gas exchange levels are different among groups. The pO_2_ levels differed between BO1 and BO2 (*p* = 0.04). Moreover, for the pCO_2_ levels, the group BO1 presented the highest concentration, while the group BO3 presented the minimum (*p* = 0.02). In the results of Table 3 for calcium, the puppies born BO1 presented the highest levels and a statistically significant difference (*p* = 0.005) in comparison to BO2, BO3 and BO5, this last group being the one which registered the lowest percentage in its levels (49.25 ± 1.77%) (Table 3).

### 3.2. Physiological Parameters According to Birth Order in Live-Born Canine Puppies from Medium-Size Female Dogs

In Table 1, in medium-size female dogs with the largest litter size (BO1–BO7), it is observed that there is no significant difference in birth order in the levels of lactate and glucose (*p* = 0.49). For the case of acid-base balance, in Table 1, it was observed that pH did not show any significant difference among the groups (*p* = 0.65) or pCO_2_ (*p* = 0.52) (Table 2). It was only observed that pO_2_ values were statistically different between BO1 (10.25 ± 0.75 mmHg) and BO6 (12.98 ± 1.09 mmHg) (*p* = 0.02) with an increase in the concentrations of pCO_2_ in BO1 (74.96 ± 2.6 mmHg). On the other hand, only BE showed differences between BO4 (−9.37 ± 0.78 mEq/L) and BO7 (−13.70 ± 1.90 mEq/L) (*p* = 0.03) (Table 3).

### 3.3. Physiological Parameters According to Birth Order in Live-Born Canine Puppies from Large-Size Female Dogs

In Table 1, no statistical changes were found regarding lactate levels among the canine neonates from large-size female dogs considering birth order (BO1–BO9) (*p* = 0.41). However, the canine neonates presented hypoglycemia immediately after the birth, except for group BO8 (82.16 ± 6.86 mg/dL), considering normal reference ranks, which reflects a drastic decrease in fetal circulation as a consequence of delivery, reducing the hepatic glycogen rapidly, thus reflecting inefficient glucose homeostasis in the newborn [40].

It is important to point out that, for blood pH, no significant difference was found among the groups (*p* = 0.33). In Table 2, the neonates that presented more alterations in pO_2_ were the puppies of BO4 (9.30 ± 0.71 mmHg) compared to BO7 (11.75 ± 0.90 mmHg) with a statistically significant difference (*p* = 0.03); likewise, BO4 presented a higher concentration of pCO_2_ (78.93 ± 2.48 mmHg), and the lowest was obtained by group BO8 (68.83 ± 4.08 mmHg) (*p* = 0.03).

### 3.4. Physiological Parameters According to Birth Order in Live-Born Canine Puppies from Female Dogs of Different Sizes

The comparison of puppies with the same birth order was evaluated with the varying size of the dam (small, medium, and large). However, it must be highlighted that it was not possible to compare all the groups due to the number of puppies within the litters.

Table 1 shows that, for lactate metabolite, there were significant differences in BO2, BO3, BO4, and BO5 (*p* < 0.05), while for glucose, only BO3 had statistical differences of *p* < 0.05 among the groups because glucose presented a minimum value of 62.17 ± 4.21 mg/dL in the large size.

On the other hand, in Table 1, the blood pH levels differed neither among the dam’s size groups, nor the birth order groups. However, in Table 2, it was observed that for the pO_2_ levels, the birth orders BO2, BO4, BO5, and BO6 showed a significant difference with the dam’s size (*p* < 0.05), as in pCO_2_, the difference was shown in groups BO2, BO3, and BO4 (*p* < 0.05). Regarding HCO_3_^−^ concentrations, there was a statistically significant difference in groups BO2 and BO4. Lastly, for EB, the difference (*p* < 0.05) was found in BO3 and BO4 (Table 1).

Moreover, in Table 3, the concentrations of calcium in the blood showed a difference (*p* < 0.05) in BO2, BO3, and BO4, while for hematocrit, again BO2, BO3, BO4, and BO5 were different (*p* < 0.05).

In Table 4, the Pearson rank test shows that, except for glucose (*p* = 0.05), the values of the physiological profile variables (pH, pCO_2_, pO_2_, Ca^2+^, Lactate, Htc, HCO_3_^−^ and BE) show a slight strength of association to birth order (BO) (*p* < 0.05) without considering the size of the mother. However, when the variable size of the mother (small, medium, and large) is introduced, as shown in Table 5, it is observed that the values of the physiological profile variables (pH, pCO_2_, pO_2_, Glucose, Ca^2+^, Lactate, HCO_3_^−^ and BE), except for Htc (*p* = 0.15), are associated with birth order only in offspring from large-sized bitches (*p* < 0.05).

Finally, in Table 6, considering the sex of the liveborn puppies’ group (LBP), the percentage of males is higher compared to females (53.23% and 46.77%). Within males, the higher percentage was observed in litters from large sized bitches (42.90%) from the total of 310 registered. Nonetheless, the X^2^ test did not show statistically significant differences (*p* > 0.05).

## 4. Discussion

Birth in all mammalian species is accompanied by a period of obligatory or transitory asphyxia in the newborn. The uterine contractions during normal parturition increase the intrauterine pressure, compromising placental perfusion and oxygenation and gas interchange. Umbilical cord blood gas analysis provides valuable information about the neonate’s condition immediately after birth in all fetuses. The results that were obtained in this study permitted the identification or the degree of alteration of the neonatal physiological blood profile according to the birth order of the pups and the dam’s size.

### 4.1. Physiological Parameters According to Birth Order in Canine Puppies from Small-Size Female Dogs

Although the uterine contractions during term labor result in a rise in intrauterine pressure, compromising placental perfusion and oxygenation, affecting gas interchange in all fetuses, the results, in general, suggest that the degree of alteration of the neonatal physiological profile is modified according to birth order. In the group of small-size dams, it was observed that the newborn puppies of BO1 presented more blood physiological alterations than those born in BO2, BO3, BO4, and BO5. This repeated impairment of gaseous exchange leads to a slight but consistent reduction in pH (7.05 ± 0.03), pO2 (11.44 ± 0.87 mmHg), and an increase in pCO2 (69.19 ± 3.02), besides presenting higher concentrations of calcium (1.72 ± 0.02 mmol/L). These variations could indicate the development of a process of intrapartum hypoxia, as referred to by Ferreiro [41] and Mota-Rojas et al. [42,43].

As stated by Uchańska et al. [44], hypoxia can cause almost 60% of neonatal deaths; the interruption of umbilical circulation during prolonged parturition or dystocia is one of the main reasons. This is similar to what was reported in other studies that mention maternal factors such as duration in the expulsion stage or the high stress of the peripartum period [32,45,46], which could result in oxygen reduction, as it has been observed in piglets [42]. However, it is important to point out that the decrease in the interchange of gasses during the delivery of these pups led to an O_2_ deficit (hypoxemia) which gave way to a rise in CO_2_ in the extracellular liquid (hypercapnia). This could conditionate to a rise in the concentration of bicarbonate (HCO_3_) which would eventually culminate in the presence of a respiratory acidosis [45]. However, the pups born in BO1 did not show a rise in HCO3 concentration; therefore, they did not show changes in blood pH, so the blood lactate and glucose values were not observed as altered.

These hemodynamic alterations in the fetus are established to enable breathing, decreasing the flow in the pulmonary vascular resistance [47]. This translates to an increase in the pulmonary vascular flow with a rise in oxygen concentration in blood and then oxygen saturation, producing the elimination of placental circulation [48]. This is why it is possible to consider that, in this study, this period of brief hypoxia results to be normal during the delivery process [49]. Only in the case of persistent hypoxia would it cause a delay in breathing and a possible metabolic acidosis, as it relates to neonatal morbidity and mortality [50]. Nevertheless, the percentage of mortality in pups of the group of small-size dams was not significant. Experimental studies with the asphyxia model confirm that the healthy fetus to be born has an impressive tolerance to hypoxemia. However, it has been reported that puppies from small-size dams present a lesser vulnerability and mortality risk than puppies from large-size dams; nevertheless, if SBP is detected, the LBP requires extra monitoring during the perinatal period [22]. Likewise, some authors have reported an influence of the birth position on the presentation of hypoxia during parturition [51]. This factor was not assessed in the present study but could be a relevant factor for further studies.

### 4.2. Physiological Parameters According to Birth Order in Canine Puppies from Medium-Size Female Dogs

For this category, group BO7 presented more acid-base, energetic and calcium balance alterations than the puppies from large-size dams. Our study states that this could be explained by the development of an intrapartum hypoxic process in the litters, especially in those born at the end of the delivery. The alteration in gas exchange can generate different degrees of hypoxia, hypercapnia, and acidosis according to the duration and severity of the interruption in the oxygen flux [52]. The delay at the beginning of the pulmonary ventilation at birth provoked a reduction in the blood’s oxygen saturation of the pups belonging to the group of medium-size dams; the persistent and higher degree of hypoxia caused a metabolic change to glycolysis with the consequent increased production of lactate, a result that can be observed when compared to the pups of small-size dams. The latter could be due to fatigue in the female dog in the expulsion of more numerous litters, in which a decrease in the number and intensity of uterine contractions could be observed, findings similar to what is reported by Mota-Rojas et al. [42] in litters of piglets. In dogs, the concentration of oxytocin and the expression of oxytocin receptors are key elements for the onset of parturition. Since this hormone maintains the synchronized uterine contractions and dilatation of the cervix to facilitate a eutocic parturition [17], abnormalities in the oxytocinergic system could extend the time of parturition and, therefore, the consequences of delayed birth. For example, Cornelius et al. [53] determined that canine pups with dystocia were 2.35 times more likely to be stillborn due to one of the leading causes of delayed parturition: uterine inertia. Further, the decrease in pH (6.97 ± 0.07) and the significant increase in the concentrations of lactate (10.00 ± 1.01 mg/dL) in this study suggest a state of metabolic acidosis [53], which could be considered to be severe, which is similar to the findings mentioned by Mota-Rojas et al. [54,55] and van Dijk et al. [56]. Moreover, prolonged hypoxia could have contributed to the low glucose levels (54.00 ± 11.88 mg/dL), predisposing toy and small breed puppies to hypoglycemia, as stated by Münnich and Küchenmeister [57]. This differs from our results, and it could be attributed to the fast consumption of the newborn’s energy reserves. Similarly, the finding of a high concentration of calcium in the blood (1.78 ± 0.06 mmol/L) suggests that the muscular activity of the puppy was increased at the moment of labor, which fostered the mobilization of the metabolite from the newborn’s bones, as pointed out in the studies made by Rydhmer et al. [58] and Mota-Rojas et al. [42].

The analysis indicates that the alterations in the metabolic profile of the late-born puppies (BO7) of medium-size female dogs in our study are a sign of the consumption of muscular glycogen, which could be due to the restriction of oxygen in the uterus, as explained by Mota-Rojas et al. [54], with the possibility that fetal suffering could also be present. This suggests that the long-term births would increase the risk of hypoxia in late-born puppies, with a more frequent Type II SBP and weak puppies that die rapidly after birth, as reported by Indrebφ et al. [59] and Münnich and Küchenmeister [57], and observed in our results with a percentage of 26.42% of intrapartum deaths Type II.

### 4.3. Physiological Parameters According to Birth Order in Neonate Canine Puppies from Large-Size Female Dogs

In this group, the reduction in base excess and the increase in pCO_2_ and lactate during the glucogenesis, and the evidence of hypoxia due to respiratory failure compared to puppies from medium-sized dams (BO6 or later) can be attributed to acidosis during birth, of metabolic and respiratory types. Acidosis can be a consequence of stress even in a normal delivery where tissue hypoxia and placentary insufficiency occur. This was mentioned by Plavec et al. [60], who reported hyperlactatemia of 12.24 ± 0.56 mmol/L and acidosis in pups from vaginal parturition due to the decreased placental circulation caused by uterine contractions. This results in a mixture of metabolic and respiratory acidosis [61] when the passage of oxygen is restricted and the carbohydrate metabolism is affected [62], shifting to anaerobic activity and the consequent accumulation of lactic acid which decreases blood pH [63]. Additionally, a hypothesis to this effect could be that an increase in the size of the products and the number of puppies in the litter could cause a higher number of uterine contractions of higher intensity, triggering a lower blood flow which reduces the oxygen supply to the fetus. This factor negatively impacts the gas interchange: the metabolites and gases of the physiological profile are, in fact, altered, as was observed in the puppies of our study. This evidence could also be promoted by amniotic liquid at the pulmonary level during the first minute of life, as reported by Vannucchi et al. [64]. Additionally, this acidosis could have increased blood calcium due to muscular mobilization [65], mainly in the puppies in the birth order BO1, BO3, BO4, and BO8 (*p* = 0.04). The alteration of the acid-base balance involves the complex interaction of several organ systems, including the brain, lungs, and liver [63]. Therefore, evaluating this parameter could help to reduce the systemic alterations that the newborn can develop.

### 4.4. Physiological Parameters According to Birth Order in Canine Puppies from Female Dogs of Different Sizes

Based on the results, it was possible to establish that the birth orders BO2, BO4, and BO7 in puppies of small and medium-size dams, and the last third of BO, presented the most maladjustments with an inadequate gas interchange. Generating hypoxia and hypercapnia due to the accumulation of carbon dioxide resulted in biochemical changes inside the neonates’ bodies, which would probably generate the death of neuronal cells and brain damage, as pointed out by Pitsawong and Panichkul [66].

According to the distribution of LP and SB type II, it is considered that, despite not observing significant differences in the dam’s size, this could be due to the litter’s size and the breed, which could be predisposing factors to stillbirths. In our research, the large size registered 56.60% of the highest percentage, with more than a half of the individuals (*n* = 53). Regarding the sex of the puppies, the percentages of small-sized male puppies obtained in the present study were close to that reported in a study analyzing the different types of parturitions (5.35% vs. 3.3%) [60]. Although the present results show a higher percentage of SB type II males than females in medium and large sizes, regardless of the sex, to date, studies report no significant association between sex and stillbirth risk in canine pups [53].

When considering the Pearson rank test and the correlations between large sized dams and the physiological profile variables (pH, pCO_2_, pO_2_, glucose, Ca^2+^, lactate, HCO_3_^−^ and BE), Mila et al. [67] also reported a significant effect of breed size on the lactate concentration of large breed puppies (1.4 mmol/L). Nonetheless, the authors concluded that the effect of the physiological moderate metabolic acidosis makes it unclear if this factor could help during the assessment of newborn puppies.

Finally, it is important to underline that alteration in the neonate’s respiratory profile could be due to the regulation of breathing, which involves a selective reduction in the consumption of oxygen during the hypoxic process, restricting the function of chemoreceptors and being capable of redistributing the blood flow to the heart, brain, diaphragm, and adrenal glands, but not to the spleen, gastrointestinal tract, skin, and kidneys. Therefore, in severe hypoxia, there would be a decrease in heart rate and an increase in intestinal motility, leading to the expulsion and aspiration of meconium and Meconium Aspiration Syndrome (MAS) and damage to the intestinal mucosa [68,69], as well as failure in other tissues that require great supplies of oxygen [70]. Other factors that compromise the viability of the canine neonate must be considered and not analyzed in this study, such as the fetus’s position at birth, the umbilical cord morphology, degree of meconium staining, and premature deliveries, which have already been reported in human medicine [71], would also have an impact in the presence of intrapartum asphyxiation in canine puppies.

## 5. Conclusions

During the eutocic delivery process, fetuses are exposed to intermittent periods of hypoxia. However, they can activate adaptive mechanisms to protect the organism from hypoxemia. The results obtained in the present study show that the size of the mother and the order of birth are risk factors for newborn puppies since they may present greater physiological blood alterations that affect adaptation to extrauterine life. It was identified that BO1 puppies of small-sized bitches, as well as BO7 puppies (last litter) of medium-sized bitches, presented more significant blood alterations. In small-sized females, this effect can be attributed to uterine morphology and activity, such as intensity, duration, or the number of contractions. Contrarily, in the last pups of medium-sized bitches, it may be due to the hypoxic process of exhaustion in the newborn due to prolonged labor time, causing weak puppies at the first minute of life. Notably, no effects were observed by birth order in puppies from large-sized female dogs, since all presented considerable blood physiological alterations, leading to intrapartum asphyxiation processes and prolonged hypoxia, affecting neonatal survival.

## Figures and Tables

**Table 1:** Mean and standard error of blood physiological profile of live-born canine puppies according to birth order and dam’s size.

Blood Traits	Dam’s	BO	BO	BO	BO	BO	BO	BO	BO	BO	Value of
	Size	1	2	3	4	5	6	7	8	9	*p*
		(Mean ± SE)	(Mean ± SE)	(Mean ± SE)	(Mean ± SE)	(Mean ± SE)	(Mean ± SE)	(Mean ± SE)	(Mean ± SE)	(Mean ± SE)	
Lactate (mg/dL)	Small *n* = 75	8.75 ± 0.44 ^a,1^	7.87 ± 0.39 ^a,2^	8.06 ± 0.41 ^a,2^	7.99 ± 0.50 ^a,2^	7.57 ± 0.73 ^a,2^	…	…	…	…	0.12
	Medium *n* = 102	9.49 0.38 ^a,1^	9.59 ± 0.39 ^a,1^	10.04 ± 0.37 ^a,1^	9.19 ± 0.41 ^a,1,2^	9.90 ± 0.42 ^a,1^	8.89 ± 0.55 ^a,1^	10.00 ± 1.016 ^a,1^	…	…	0.49
	Large *n* = 133	9.77 ± 0.32 ^a,1^	10.04 ±0.34 ^a,1^	10.44 ± 0.36 ^a,1^	10.05 ± 0.36 ^a,1^	9.55 ± 0.355 ^a,1^	9.50 ± 0.377 ^a,1^	10.45 ± 0.45 ^a,1^	10.66 ± 0.58 ^a^	10.44 ± 0.84 ^a^	0.41
	Value of *p*	*p* > 0.05	*p* < 0.05	*p* < 0.05	*p* < 0.05	*p* < 0.05	*p* > 0.05	*p* > 0.05			
Glucose (mg/dL)	Small *n* = 75	66.21 ± 5.14 ^a,1^	75.69 ± 4.64 ^a,1^	70.85 ± 4.88 ^a,1,2^	74.92 ± 5.90 ^a,1^	78.49 ± 8.54 ^a,1^	…	…	…	…	0.20
	Medium *n* = 102	70.47 ± 4.44 ^a,1^	65.35 ± 4.60 ^a,1^	74.18 ± 4.40 ^a,1^	71.52 ± 4.89 ^a,1^	69.18 ± 5.00 ^a,1^	78.84 ± 6.46 ^a,1^	54.00 ± 11.88 ^a,1^	…	…	0.49
	Large *n* = 133	74.49 ± 3.81 ^a,1^	65.59 ± 4.08 ^b,1^	62.17 ± 4.21 ^b,2^	65.53 ± 4.23 ^b,1^	71.77 ± 4.16 ^a,b,1^	72.16 ± 4.41 ^a,b,1^	74.29 ± 5.33 ^a,b,1^	82.16 ± 6.86 ^a^	74.00 ± 9.91 ^a,b^	0.02
	Value of *p*	*p* > 0.05	*p* > 0.05	*p* < 0.05	*p* > 0.05	*p* > 0.05	*p* > 0.05	*p* > 0.05			
pH	Small *n* = 75	7.05 ± 0.03 ^a,1^	7.08 ± 0.03 ^a,1^	7.10 ± 0.03 ^a,1^	7.08 ± 0.03 ^a,1^	7.05 ± 0.05 ^a,1^	…	..	..	…	0.53
	Medium *n* = 102	7.08 ± 0.02 ^a,1^	7.068 ± 0.03 ^a,1^	7.06 ± 0.029 ^a,1^	7.06 ± 0.03 ^a,1^	7.08 ± 0.03 ^a,1^	7.07 ±0.04 ^a,1^	6.97 ± 0.07 ^a,1^	…	…	0.65
	Large *n* = 133	7.10± 0.02 ^a,1^	7.07 ± 0.02 ^a,1^	7.04 ± 0.02 ^a,1^	7.04 ± 0.02 ^a,1^	7.03 ± 0.02 ^a,1^	7.07 ± 0.29 ^a,1^	7.05 ± 0.03 ^a,1^	7.12 ± 0.04 ^a^	7.14 ± 0.06 ^a^	0.33
	Value of *p*	*p* > 0.05	*p* > 0.05	*p* > 0.05	*p* > 0.05	*p* > 0.05	*p* > 0.05	*p* > 0.05			
HCO_3_^−^ (mmol/L)	Small *n* = 75	19.42 ± 0.49 ^a,1^	20.36 ± 0.44 ^a,1^	19.97 ± 0.47 ^a,1^	20.18 ± 0.56 ^a,1^	19.61 ± 0.82 ^a,1^	…	…	…	…	0.35
	Medium *n* = 102	19.77 ± 0.42 ^a,1^	19.71 ± 0.44 ^a,1,2^	19.75 ± 0.42 ^a,1^	19.81 ± 0.47 ^a,1^	19.21 ± 0.48 ^a,1^	19.78 ± 0.62 ^a,1^	18.75 ± 1.14 ^a,1^	…	…	0.61
	Large *n* = 133	19.64 ± 0.36 ^a,1^	18.94 ± 0.39 ^a,2^	19.20 ± 0.40 ^a,1^	18.75 ± 0.40 ^a,2^	19.27 ± 0.40 ^a,1^	19.12 ± 0.42 ^a,1^	18.86 ± 0.51 ^a,1^	19.01 ± 0.66 ^a^	19.00 ± 0.95 ^a^	0.03
	Value of *p*	*p* > 0.05	*p* < 0.05	*p* > 0.05	*p* < 0.05	*p* > 0.05	*p* > 0.05	*p* > 0.05			
BE (mEq/L)	Small *n* = 75	−10.23 ± 0.82 ^a,1^	−9.58 ± 0.74 ^a,1^	−9.09 ± 0.78 ^a,2^	−9.15 ± 0.94 ^a,2^	−9.32 ± 1.37 ^a,1^	…	…	…	…	0.16
	Medium *n* = 102	−9.90 ± 0.71 ^a,b,1^	−10.91 ± 0.73 ^a,b,1^	−10.95 ± 0.70 ^a,b,1,2^	−9.37 ± 0.78 ^b,2^	−10.51 ± 0.80 ^a,b,1^	−10.01 ± 1.03 ^a,b,1^	−13.70 ± 1.90 ^a,1^	…	…	0.03
	Large *n* = 133	−10.48 ± 0.612 ^a,1^	−10.68 ± 0.65 ^a,1^	−11.82 ± 0.67 ^a,1^	−11.85 ± 0.68 ^a,1^	−10.97 ± 0.66 ^a,1^	−11.28 ± 0.72 ^a,1^	−10.08 ± 0.85 ^a,1^	−12.05 ± 1.10 ^a^	−11.96 ± 1.58 ^a^	0.41
	Value of *p*	*p* > 0.05	*p* > 0.05	*p* < 0.05	*p* < 0.05	*p* > 0.05	*p* > 0.05	*p* > 0.05			

SE: Standard error. BO: birth order; ^a, b^ indicate significant difference *p* < 0.05 among rows in the same dam’s size, depending on birth order; ^1, 2^ indicate difference among columns in the same birth order, depending on dam’s size; Dam’s size: according to the category: Small (<10 kg), Medium (11–25.0 kg), Large (26–45 kg); *n* = number of newborn puppies according to the dam’s size; ANOVA, Mixed General Linear Model, Tukey.

**Table 2:** Mean and standard error of blood gas exchange of live-born canine puppies according to birth order and dam’s size.

Blood Traits	Dam’s	BO	BO	BO	BO	BO	BO	BO	BO	BO	Value of
	Size	1	2	3	4	5	6	7	8	9	*p*
		(Mean ± SE)	(Mean ± SE)	(Mean ± SE)	(Mean ± SE)	(Mean ± SE)	(Mean ± SE)	(Mean ± SE)	(Mean ± SE)	(Mean ± SE)	
pO_2_ (mmHg)	Small *n* = 75	11.44 ± 0.87 ^b,1^	13.32 ± 0.78 ^a,1^	12.54 ± 0.82 ^a,b,1^	12.86 ± 0.99 ^a,b,1^	12.47 ± 1.44 ^a,b,1,2^	----	---	---	---	0.04
	Medium *n* = 102	10.25 ± 0.75 ^c,1^	10.92 ± 0.78 ^a,b,c,2^	10.78 ± 0.76 ^a,b,c,1,2^	11.80 ± 0.82 ^a,b,c,1^	12.47 ± 0.84 ^b,1^	12.98 ± 1.09 ^a,1^	9.00 ± 2.01 ^a,b,c,1^	---	---	0.02
	Large *n* = 133	10.98 ± 0.64 ^a,b,1^	10.34 ± 0.69 ^a,b,2^	9.64 ± 0.71 ^a,b,2^	9.30 ± 0.71 ^b,2^	9.99 ± 0.70 ^a,b,2^	9.95 ± 0.74 ^a,b,2^	11.75 ± 0.90 ^a,1^	11.83 ± 1.62 ^a,b^	8.88 ± 1.67 ^a,b^	0.0
	Value of *p*	*p* > 0.05	*p* < 0.05	*p* > 0.05	*p* < 0.05	*p* < 0.05	*p* < 0.05	*p* > 0.05			
pCO_2_ (mmHg)	Small *n* = 75	69.19 ± 3.02 ^a,1^	63.72 ± 2.73 ^a,b,2^	62.05 ± 2.87 ^b,2^	65.81 ± 3.47 ^a,b,2^	63.93 ± 5.02 ^a,b,1^	…	…	…	…	0.02
	Medium *n* = 102	74.96 ± 2.61 ^a,1^	73.33 ± 2.70 ^a,1^	74.42 ± 2.58 ^a,1^	70.27 ± 2.87 ^a,2^	74.28 ± 2.94 ^a,1^	70.31 ± 3.80 ^a,1^	77.00 ± 6.98 ^a,1^	…	…	0.52
	Large *n* = 133	74.90 ±2.24 ^a,b,1^	74.11 ± 2.40 ^a,b,1^	74.80 ± 2.47 ^a,b,1^	78.93 ±2.48 ^a,1^	74.41 ± 2.44 ^a,b,1^	75.33 ± 2.59 ^a,b,1^	75.13 ± 3.13 ^a,b,1^	68.83 ± 4.03 ^b^	71.25 ± 5.82 ^a,b^	0.03
	Value of *p*	*p* > 0.05	*p* < 0.05	*p* < 0.05	*p* < 0.05	*p* > 0.05	*p* > 0.05	*p* > 0.05			

SE: Standard error. BO: birth order; ^a, b, c^ indicate significant difference *p* < 0.05 among rows in the same dam’s size, depending on birth order; ^1, 2^ indicate difference among columns in the same birth order, depending on dam’s size; Dam’s size: according to the category: Small (<10 kg), Medium (11–25.0 kg), Large (26–45 kg); *n* = number of newborn puppies according to the dam’s size; ANOVA, Mixed General Linear Model, Tukey.

**Table 3:** Mean and standard error of calcium concentrations and hematocrit in live-born canine puppies according to birth order and dam’s size.

Blood Traits	Dam’s	BO	BO	BO	BO	BO	BO	BO	BO	BO	Value of
	Size	1	2	3	4	5	6	7	8	9	*p*
		(Mean ± SE)	(Mean ± SE)	(Mean ± SE)	(Mean ± SE)	(Mean ± SE)	(Mean ± SE)	(Mean ± SE)	(Mean ± SE)	(Mean ± SE)	
Ca^2+^ (mmol/L)	Small *n* = 75	1.72 ± 0.02 ^a,1^	1.63 ± 0.02 ^b,2^	1.60 ± 0.02 ^b,2^	1.65 ± 0.03 ^a,b,2^	1.67 ± 0.04 ^a,b1^	…	…	…	…	0.005
	Medium *n* = 102	1.73 ± 0.02 ^a,1^	1.71 ± 0.02 ^a,1^	1.75 ± 0.02 ^a,1^	1.69 ±0.02 ^a,2^	1.72 ± 0.02 ^a,1^	1.71 ± 0.03 ^a,1^	1.78 ± 0.06 ^a,1^	…	…	0.00
	Large *n* = 133	1.71 ± 0.02 ^b,1^	1.76 ± 0.02 ^a,b,1^	1.79 ± 0.02 ^a,1^	1.77 ±0.02 ^a,1^	1.75 ± 0.02 ^a,b,1^	1.72 ± 0.02 ^b,1^	1.77 ± 0.03 ^a,b,1^	1.70 ± 0.03 ^b^	1.72 ± 0.05 ^a,b^	0.04
	Value of *p*	*p* > 0.05	*p* < 0.05	*p* < 0.05	*p* < 0.05	*p* > 0.05	*p* > 0.05	*p* > 0.05			
Htc (%)	Small *n* = 75	53.76 ± 1.06 ^a,1^	51.24 ± 0.96 ^b,2^	51.40 ± 1.01 ^b,2^	52.76 ± 1.22 ^a,b,2^	49.25 ±1.77 ^b,2^	…	…	…	…	0.02
	Medium *n* = 102	53.53 ± 0.92 ^a,1^	53.60 ± 0.95 ^a,1,2^	54.04 ± 0.91 ^a,1^	52.72 ± 1.01 ^a,1,2^	55.31 ± 1.04 ^a,1^	54.58 ± 1.34 ^a,1^	57.10 ± 2.47 ^a,1^	…	…	0.39
	Large *n* = 133	53.94 ± 0.79 ^a,1^	54.51 ± 0.85 ^a,1^	54.87 ± 0.87 ^a,1^	55.41 ± 0.88 ^a,1^	54.78 ± 0.86 ^a,1^	53.22 ± 0.91 ^a,1^	53.41 ± 1.10 ^a,1^	53.60 ± 1.42 ^a^	51.08 ± 2.06 ^a^	0.49
	Value of *p*	*p* > 0.05	*p* < 0.05	*p* < 0.05	*p* < 0.05	*p* < 0.05	*p* > 0.05	*p* > 0.05			

SE: Standard error. BO: birth order; ^a, b^ indicate significant difference *p* < 0.05 among rows in the same dam’s size, depending on birth order; ^1, 2^ indicate difference among columns in the same birth order, depending on dam’s size; Dam’s size: according to the category: Small (<10 kg), Medium (11–25.00 kg), Large (26–45 kg); *n* = number of newborn puppies according to the dam’s size; ANOVA, Mixed General Linear Model, Tukey.

**Table 4:** Correlations between birth order (BO) and blood physiological profile in live-born canine puppies.

(y)	(x)	*r* Value	*p* Value
BO	pH	−0.19	*p* = 0.00
	pCO_2_ (mmHg)	0.17	*p* = 0.00
	pO_2_ (mmHg)	−0.15	*p* = 0.00
	Glucose (mg/dL)	−0.10	*p* = 0.05
	Ca^2+^ (mmol/L)	0.20	*p* = 0.00
	Lactate (mg/dL)	0.24	*p* < 0.00
	Htc (%)	0.18	*p* = 0.00
	HCO_3_^−^ (mmol/L)	−0.21	*p* = 0.00
	BE (mEq/L)	0.24	*p* < 0.00

Dependent variable (y), birth order (BO); independent variable (x), dam size, blood physiological profile. The data are presented as a correlation coefficient (*r* value); *n* = 310; *n* = number of newborn puppies.

**Table 5:** Correlations between birth order (BO) and blood physiological profile according to dam’s size.

(y)	(x)	Dam’s Size		
		Small *n* = 75	Medium *n* = 102	Large *n* = 133
BO	pH	−0.015 *p* = 0.89	−0.04 *p* = 0.65	−0.25 *p* = 0.00
	pCO_2_ (mmHg)	−0.06 *p* = 0.59	−0.02 *p* = 0.83	0.20 *p* = 0.02
	pO_2_ (mmHg)	0.03 *p* = 0.79	0.13 *p* = 0.17	−0.20 *p* = 0.01
	Glucose (mg/dL)	0.09 *p* = 0.43	−0.12 *p* = 0.20	−0.17 *p* = 0.04
	Ca^2+^ (mmol/L)	−0.07 *p* = 0.53	0.01 *p* = 0.88	0.18 *p* = 0.03
	Lactate (mg/dL)	−0.056 *p* = 0.63	0.02 *p* = 0.82	0.28 *p* = 0.00
	Htc (%)	−0.14 *p* = 0.21	0.12 *p* = 0.22	0.12 *p* = 0.15
	HCO_3_^−^ (mmol/L)	−0.07 *p* = 0.54	−0.08 *p* = 0.42	−0.21 *p* = 0.01
	BE (mEq/L)	0.13 *p* = 0.25	−0.03 *p* = 0.70	−0.27 *p* = 0.00

Dependent variable (y), birth order (BO); independent variable (x), dam size, blood physiological profile. The data are presented as a correlation coefficient (*r* value). Dam’s size: according to the category: Small (<10 kg), Medium (11–25.00 kg), Large (26–45 kg). *n* = number of newborn puppies according to the dam’s size.

**Table 6:** Frequency and percentage of canine liveborn puppies (LBP) by sex according to the size of the bitch.

Dam’s size	Female *n =* 145 (46.77%)	Male *n =* 165 (53.23%)	Total *n =* 310
	LBP	LBP	
Small	38 (46.67%) ^a,1^	37 (41.33%) ^a,1^	75 (24.20%)
Medium	49 (45.10%) ^a,1^	53 (41.18%) ^a,1^	102 (32.90%)
Large	58 (39.10%) ^a,1^	75 (38.35%) ^a,1^	133 (42.90%)

^a, b^ indicate significant difference (*p* < 0.05) among columns in the same sex, to the *x^2^* test; ^1, 2^ indicate significant difference (*p* < 0.05) among rows in the same dam’s size, to the *x^2^* test; LBP: liveborn puppies; dam’s size = according to the category: Small (<10 kg), Medium (11–25.0 kg), Large (26–45 kg).

## Data Availability

Data is contained within the article.

## Abbreviations

pCO_2_	partial carbon dioxide saturation
pO_2_	partial oxygen saturation
O_2_	oxygen
BE	base excess
HCO_3_^−^	bicarbonate
Ca^++^	calcium
Htc	hematocrit
